# Development of an Ultrasonic Method for the Quality Control of Polyethylene Tanks Manufactured Using Rotational Molding Technology

**DOI:** 10.3390/polym15102368

**Published:** 2023-05-19

**Authors:** Vitaliy Tyukanko, Alexandr Demyanenko, Vladislav Semenyuk, Antonina Dyuryagina, Dmitry Alyoshin, Roman Tarunin, Vera Voropaeva

**Affiliations:** Department of Chemistry and Chemical Technology, Manash Kozybayev North Kazakhstan University, Petropavlovsk 150000, Kazakhstan; demianenkoav@mail.ru (A.D.); evdimid@mail.ru (V.S.); adyuryagina@inbox.ru (A.D.); dvaleshin@ku.edu.kz (D.A.); ratarunin@ku.edu.kz (R.T.); vera0805023@inbox.ru (V.V.)

**Keywords:** rotomolding, rotational molding, polyethylene tanks, ultrasonic inspection, nondestructive testing, ultrasound in plastics, polymer quality control, higher harmonics, spectrum analysis

## Abstract

Tanks made of three different brands of rotational polyethylene (DOW, ELTEX, and M350) with three degrees of sintering (normal sintering (NS), incomplete sintering (ICS), and thermally degraded sintering (TDS)) and three thicknesses (7.5 mm, 8.5 mm, and 9.5 mm) were explored. It was found that the thickness of the walls of the tanks did not have a statistically significant effect on the parameters of the ultrasonic signal (USS). An increase in temperature caused a decrease in the USS parameters. According to the temperature coefficient of stability, the ELTEX brand of plastic can be distinguished (from DOW and M350). The ICS degree of the sintering of the tanks was revealed from a significantly lower amplitude of the bottom signal, compared with NS and TDS degree samples. By analyzing the amplitude of the third harmonic of the ultrasonic signal (β), three degrees of the sintering of containers NS, ICS, and TDS were revealed (with an accuracy of about 95%). Equations β = *f*(T, PIAT) were derived for each brand of rotational polyethylene (PE), and two-factor nomograms were constructed. Based on the results of this research, a method for the ultrasonic quality control of polyethylene tanks manufactured using rotational molding was developed.

## 1. Introduction

Rotational molding (RM), also known as rotomolding (RM), is a high-temperature, low-pressure, low-shear process for the production of hollow plastic products [1,2,3]. RM is currently a rapidly growing sector of the polymer processing industry. Products obtained via the rotational molding of plastics are characterized by a long service life, chemical resistance, and low cost [4,5,6]. At present, the development of new formulations based on various polymers [7,8,9,10] and fillers for rotational molding is being carried out at an accelerated pace; compositions with clay [11,12], quartz [13], halloysite [14], glass fiber or powder [15,16,17], copper slag [18], or graphite [19,20] have also been proposed.

In rotational molding, the raw material is distributed over the inner surface of the cavity of the rotating mold, and the simultaneous heating of the mold causes it to melt with the formation of a thin coating in the form of a shell [21,22,23,24]. In the second stage of the process, liquid bridges are formed between the plastic particles to be fused, retaining microscale air bubbles, which should disappear in the subsequent compaction stage, and all this takes place at high temperatures above the melting point of polyethylene [25]. If these microbubbles are not eliminated, the manufactured tanks will have low impact strength and other mechanical properties [26]. A previous study [27] revealed that the size and number of microbubbles are significantly affected by the size of the filler particles [28,29], the heating rate of the mold, and the peak air temperature in the mold (PIAT) [1,3]. For some applications, products with a foamed product used in their wall structure are specially obtained [5,30,31]. However, when the product is overheated (it is held in the oven for more than the permissible time), the thermo-oxidative degradation of polyethylene (PE) occurs in the mold, leading to a significant deterioration in its mechanical properties [2,12,32,33,34]. This effect was also revealed for recycled PE [35,36,37,38,39,40,41]. The impact strength of the finished tanks is the most important characteristic for evaluating the quality of a product. It is determined, as a rule, by the conditions of the technological process [21,42] and the composition of the processed raw materials [43,44,45,46,47,48,49,50,51]. Therefore, in the RM industry, the issue of the precise control of the material sintering process is acute; currently, it is mainly carried out by monitoring PIAT.

However, due to the relatively high cost (the price of a tank can be thousands of dollars) and very significant consequences for the environment, human health, and the reputation of the company, in the case of the destruction of tanks and the ingress of toxic pesticides into the soil and poisoning of groundwater, the problem of the continuous 100% quality control of all tanks for storing liquid mineral fertilizers is very relevant. Currently, in order to maintain a high level of quality of manufactured tanks, some rotomolders check one or two of each batch via destructive control. This control method is inconvenient for two reasons. Firstly, it is a costly process. Secondly, by applying random inspection, there is still a small probability of manufacturing a defective product due to a critical equipment failure (thermocouples, telemetry, burners, etc.); in such cases, the product may conditionally be considered of high quality, but in reality, it will have significantly lower mechanical properties (yield limit and impact resistance), which is often unacceptable. Some studies [52,53] have described methods for assessing the quality of PE products by controlling the modulus of elasticity, hardness, and viscosity of the material. The thermal degradation of the material causes an increase in the viscosity of polyethylene, which can be detected via melt flow analysis [54] or the geometry of parallel plates [55]. Another study [56] described the use of Fourier transform infrared spectroscopy (FT-IR) to identify oxidation products on the surface of products manufactured via RM. Additionally, X-ray diffraction [57], Raman scattering spectroscopy [58], and X-ray microtomography [59] were used to assess changes in the PE crystal morphology for signs of destruction. In [59], IR spectroscopy revealed that the thermal-oxidative degradation of PE parts manufactured via RM mainly occurs on the inner surfaces (because they interact with heated air, whereas the outer layers are shielded due to their contact with the mold surface). In real production conditions, the inner surface of the product is inaccessible, and the outer surface is not informative due to the absence of thermal-oxidative degradation.

However, none of these control methods is applicable in the RM industry to ensure the quality of mass-produced products due to the high cost of the equipment and the impossibility of integrating it into a continuous technological process. Therefore, in order to increase the efficiency of the technological process and reduce the cost of identifying defective products, the use of non-destructive testing methods becomes relevant. As an alternative to destructive testing methods and expensive scientific instruments, ultrasonic testing methods (hereinafter referred to as ultrasonic inspection) for PE were considered, comparing the acoustic properties of different materials at different temperatures and different internal stress [60,61]. Conventional ultrasonic inspection is very effective for measuring the density of polyolefins and relating this property to changes in the velocity or attenuation of the sound wave propagating through the sample [62,63,64]. In [65], the ultrasonic detection of incomplete PE sintering was developed by analyzing the amplitude of the bottom signal. It was not possible to determine the thermal destruction of PE due to the equivalence of the bottom signal amplitudes for both normal and overheated samples. Gomes et al. [66,67,68,69] highlighted the fundamental possibility of determining the properties of microbubbles in the walls of products by analyzing the spectral amplitude of ultrasonic signals. At the same time, an increase in amplitude as PIAT increased led to a decrease in attenuation caused by a decrease in the content of microbubbles in products. In addition, it was shown that as PIAT increased (and, consequently, the thermal destruction of PE occurred), the amplitude of higher harmonics in the ultrasonic spectrum increased. However, the given PLS model, which showed the possibility of detecting both products containing microbubbles (incomplete sintering) and those subjected to thermal destruction (thermally degraded samples), was not entirely correct. This model had a large error in determining the degree of quality of products, the adequacy of the model ranged from 58.6% to 80.3%, which is not acceptable in the industry. However, Gomes et al.’s studies have three shortcomings that do not allow for the use of their results in the industry. First, only thin PE plates, with only 2 mm thickness, were investigated. However, liquid fertilizer storage tanks are typically 8 to 12 mm thick. Therefore, the factor of influence on the ultrasonic characteristics of the wall thickness was not taken into account in these studies. The second shortcoming in these studies is that the equipment used was assembled from separate blocks. However, plastic manufacturing facilities rarely employ experienced engineers with skills related to National Instruments hardware and software platforms, so standard industrial ultrasonic flaw detectors should be used whenever possible. Thirdly, when integrating ultrasonic inspection into the existing technological process, measurements should be carried out in hot molds and tanks; however, the factor of the influence of temperature on the ultrasonic characteristics of products was not taken into account in these studies. The importance of research on the influence of temperature on ultrasonic characteristics is confirmed by the results presented in [70,71].

Therefore, in this study, we developed an industrial method for monitoring the quality of RM-manufactured tanks for storing liquid mineral fertilizers and pesticide solutions via ultrasonic inspection. In this case, two technological factors (thickness and temperature) and their mutual influence on the ultrasonic characteristics of products were taken into account. Ultrasonic spectrometry is indeed a breakthrough technology for the quality control of RM plastic parts. However, considering the conditions of production, when the price of defective goods is quite high, to preserve the reputation of the enterprise, as well as from an economic point of view, it is necessary to rely on multivariate analysis. In addition to the spectrometric data of the ultrasonic signal, this type of analysis includes other parameters available both in the direct and indirect control and measurement processes of flaw detection [72,73].

## 2. Materials and Methods

### 2.1. Materials

Three brands of rotational polyethylene were used as a matrix.:

(1) Linear low-density polyethylene (LLDPE) “DOWLEX 2629UE” hexene (hereinafter “DOW”) was used as a matrix, with a density of 935 kg/m^3^ and a melt flow index (MFI) of (190 °C/2.16 kg) 0.004 kg/10 min from Dow Chemical Company (Dow Europe GmbH, Horgen, Switzerland);
(2) Linear low-density polyethylene (LLDPE) “M3504DXP” octene (hereinafter “M350”) was used as a matrix, with a density of 935 kg/m^3^ and a melt flow index (MFI) of (190 °C/2.16 kg) 0.004 kg/10 min from SCG ICO Polymers Co., Ltd. (Bangkok, Thailand);
(3) Linear low-density polyethylene (LLDPE) “ELTEX HD3850UA” hexene (hereinafter “ELTEX”) was used as a matrix, with a density of 9381 kg/m^3^ and a melt flow index (MFI) of (190 °C/2.16 kg) 0.0044 kg/10 min from INEOS (London, UK).

VYNAMON Green 600734 (PG7) from Heubach (Langelsheim, Germany) was used as a pigment.

The separating agent was fluoroplastic varnish with molybdenum disulfide MODENGY^®^ 1014 (MODENGY, Bryansk, Russia).

### 2.2. Sample Preparation

**Figure 1:**
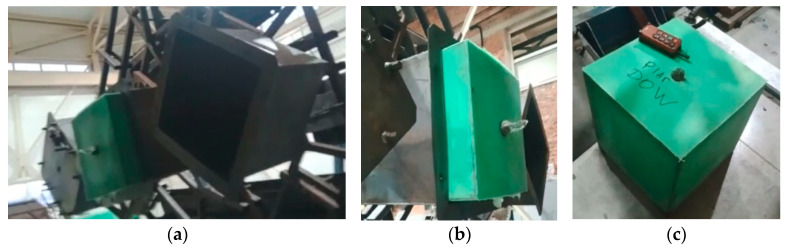
Sample molds (**a**), sample extraction (**b**), and finished sample (**c**).

The rotational molding process was carried out on a single-arm shuttle rotational molding machine, model FD4.0, manufactured by Yantai Fangda Rotational Molding Co., Ltd. (Yantai, China), with two rotation axes, equipped with three cubic steel molds. After introducing a measured amount of milled polymer and pigment, the sealed form was introduced into the heating chamber of the machine, preheated to 300 °C. The mold was kept in rotation for 25–35 min at 300 °C (heat cycle). The internal air temperature (IAT) was controlled using a thermocouple through the mold vent during the entire process. The molds were heated to the required values (from 170 to 245 °C) of the maximum internal air temperature (PIAT), after which the heating was stopped, and the cooling process began. When the IAT was reduced to 90 °C, the samples were removed from the mold (Figure 1b). During the rotational molding process (heating and cooling), the rotation speed rates for the major (arm speed) and minor (plate speed) axes were set to 5 and 9 rpm. The mold was a steel cube with dimensions of 500 mm × 500 mm × 500 mm. The shape of the molded samples and their extraction are shown in Figure 1.

To obtain samples of different thicknesses (9.5 mm, 8.5, and 7.5 mm), three cubes of 500 mm × 500 mm × 500 mm were fabricated, with a material mass of 13 kg, 11.6, and 10.2 kg. The material used was a mixture of polyethylene powder and pigment, which was mixed before molding for 5–10 min using a mixer, model VCG-150, manufactured by Yantai Fangda Rotational Molding Co., Ltd. (Yantai, China).

Based on the results of mechanical tests out of 27 samples, 9 (No. 4, 5, 6, 13, 14, 15, 22, 23, and 24 in Table 1) were selected, which were recognized as usable samples (hereinafter US) and normal sintering (hereinafter referred to as NS). Their specific mean failure energy (MFE_sp._) was more than 40 J/mm, and the density was not less than 0.9395 g/cm^3^. Nine samples with minimal PIAT (No.1, 2, 3, 10, 11, 12, 19, 20, and 21 in Table 1) were recognized as wrong (WR) and incomplete sintering (hereinafter ICS), since their specific mean failure energy (MFE_sp._) was less than 35 J/mm, and the density was less than 0.9395 g/cm^3^. Nine samples with maximum PIAT (No. 7, 8, 9, 16, 17, 18, 25, 26, and 27 in Table 1) were recognized as wrong and thermally degraded samples (hereinafter TDS) since their specific mean failure energy (MFE_sp._) was less than 40 J/mm, and the density was more than 0.9395 g/cm^3^.

### 2.3. Ultrasonic Inspection Methods

The following parameters were determined during the ultrasonic inspection of the samples:

(1) The amplitude of the bottom signal (A, %);
(2) The relative speed of the ultrasonic signal in the plastic test samples (RV, r.u.s.);
(3) The temperature coefficient of stability (TCS, %);
(4) The amplitude factor of the third harmonic (β, r.u.a.).

**Figure 2:**
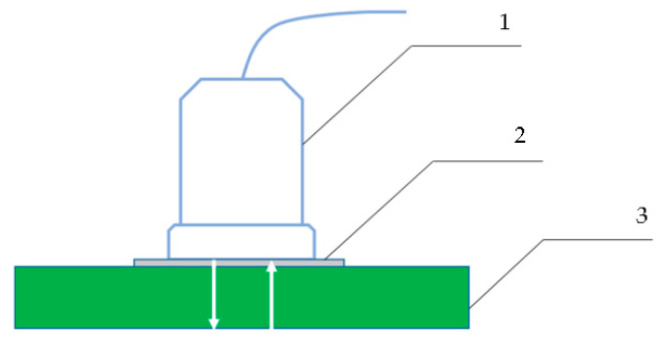
Echo-pulse method of ultrasonic flaw detection: 1—ultrasonic transducer; 2—a layer of glycerin; 3—experimental sample of plastic with a thickness of 7.5/8.5/9.5 mm.

**Figure 3:**
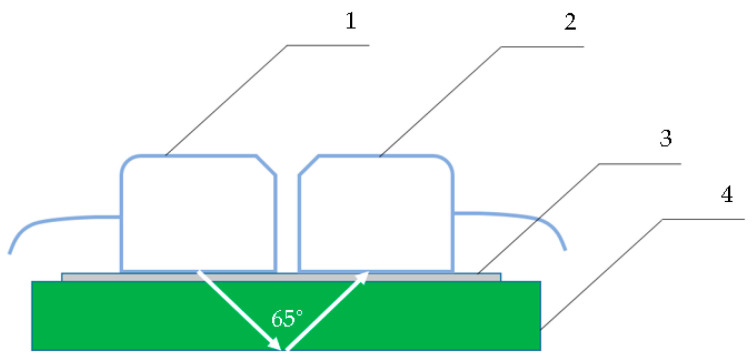
Mirror-shadow method of flaw detection: 1—inclined sensor (piezoelectric transducers) with transmitter function; 2—inclined sensor (piezoelectric transducer) with receiver function; 3—a layer of glycerin; 4—experimental sample of plastic with a thickness of 7.5/8.5/9.5 mm.

An industrial ultrasonic flaw detector UCD 60 from Kropus (Moscow, Russia) was used in the tests. To register the A, RV, and TCS parameters, the echo-pulse method was used (Figure 2), and to obtain the β of the bottom signal, the mirror-shadow method was used (Figure 3).

In the echo-pulse method, a layer of glycerin was preliminarily applied to the surface of the test plastic sample in order to improve the quality of the ultrasonic signal transmission. A piezoelectric transducer was placed on the surface treated with glycerin. Using the flaw detector, an electric pulse with an amplitude of 200 V, a filling frequency of 2.5 MHz, and a repetition rate of 20 Hz was applied to the piezoelectric transducer. After reflection from the opposite plastic wall, the bottom signal was recorded on the screen of the flaw detector, which is a key informative parameter.

To create different temperature conditions of the environment, the test samples of the PE of different brands were placed for 4 h in an ShS-80-01 SPU drying oven. The selected period of time guarantees the high-quality heating of all plastic layers, taking into account different brands, thicknesses, and degrees of sintering quality. To assess the dependence of the above optimization parameters on temperature, four temperature values were chosen: 20 °C, 40 °C, 60 °C, and 80 °C. The choice of these temperature values is associated with the peculiarity of the cooling of PE products manufactured using rotational molding. After removing the product from the mold, it is recommended to perform ultrasonic testing at a temperature of PE not higher than 80 °C.

The amplitude of the ultrasonic signal (A) in the time domain was determined as the percentage of overlap between the flaw detector display and the bottom signal (%). In order to optimally display the signal on the display, the internal amplifier of the flaw detector was set to 20 dB. The relative ultrasonic signal propagation velocity (RV) was determined using the Sa coefficient, which was taken into account as an indicator of the thickness of the product under the optimal temperatures of the production shop (18–20 °C). In the database of the results of the experiment, the speed was fixed based on a relative indicator, namely the relative unit of speed (r.u.s), and was determined with an accuracy of hundredths. The value “1.00 r.u.s” is defined as the speed at 20 °C. With a change in the Sa parameter of the UCD-60 flaw detector relative to the value at a temperature of 20 °C due to heating the plastic to other temperatures, the velocity index is calculated according to Equation (1) as follows:

(1) $$RV=\frac{Sa}{S20}$$

where S20 is the value of the Sa parameter at 20 °C.

The temperature coefficient of stability (TCS) was determined indirectly. Preliminarily, we measured all the amplitude values of the bottom signal under different heating temperatures of PE. Then, the average value A was determined. Finally, the TCS was calculated using Equation (2) as follows:

(2) $$TCS=\frac{A_{max}}{0.25\cdot (A_{1}+A_{2}+A_{3}+A_{4})}\cdot 100\%$$

where A_max_ is the maximum deviation of the bottom signal amplitude from the arithmetic mean, and A_i_ is the amplitude of the bottom signal under conditions of different temperatures of polyethylene plastic.

With the mirror-shadow method for determining the amplitude coefficient of the third harmonic (β) of an ultrasonic signal, two inclined sensors were used. The sensors were placed on one surface of the plastic, as shown in Figure 3. The distance between the input points of sensors along one plane was 17 mm. One inclined transducer acted as an ultrasonic transducer–transmitter, and the second performed the function of a receiver (Figure 3). For this control method, a signal was used with an amplitude of 200 V, a filling frequency of 3.75 MHz, a pulse repetition rate of 20 Hz, and an input angle of 65°. The inclined sensors with an angle of 65° are included in the standard supply of UCD 60. Thus, the possibility of using UCD 60 with a complete series of standard sensors to provide the quality control of rotary polyethylene was investigated. The spectrum width for the third harmonic was in the range of 7.8–13.5 MHz. The internal amplifier of the flaw detector was set to 60 dB. The β parameter was determined using an indirect measurement method. The spectrum function was set on the UCD 60 flaw detector display. On the spectral coordinate grid of the flaw detector display on the amplitude axis Oy, 10 cells were selected. Each cell had five divisions with a step of 0.02 units. The amplitude of the first harmonic (range 3.4–3.9 MHz) always filled 10 cells or 50 divisions of the spectrometric scale, and the amplitude of the third harmonic was counted from the cell divisions on the display. The calculation of β was carried out according to Equation (3) as follows:

(3) $$\beta =\frac{U_{3}}{U_{1}}$$

where β is the amplitude coefficient with the unit of measurement r.u.a (the relative unit of amplitude); U_1_ is the amplitude of the first harmonic (50 divisions); and U_3_ is the amplitude of the third harmonic (number of divisions).

In the absence of the amplitude of the third harmonic on the spectrometric scale, the noise level (NN) was determined. The noise level was also counted by the number of divisions.

### 2.4. Methods of Mechanical Testing

**Figure 4:**
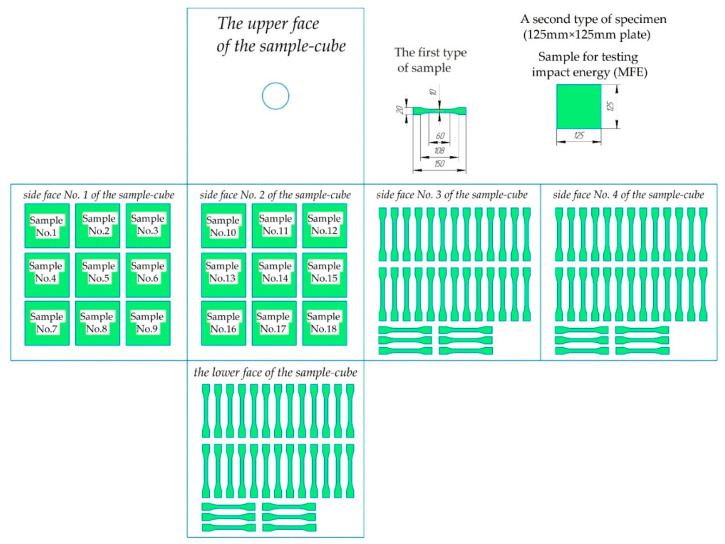
Distribution of test samples along the faces of a rotating PE cube.

From the resulting rotating 500 mm × 500 mm × 500 mm PE cubes, two types of specimens for mechanical testing (Figure 4) were cut.

The first type of sample (upper right) was used to determine the tensile properties of the material, i.e., the modulus of elasticity involving the flexural modulus (MPa), the yield strength tensile stress (yield) (MPa), and the maximum tensile stress (break) (MPa), in accordance with ISO 527-2:2012, at a speed of 5 m/min. Tensile tests were carried out on a testing machine from Haida International Equipment Co., Ltd., HD-B617-S (Dongguan, China).The samples for mechanical testing were tested at various plus temperatures (20 ± 2) °C; (40 ± 2) °C; (60 ± 2) °C; and (80 ± 2) °C. Their holding time in the oven (to equalize the temperature in the plastic) was 24 h. The resulting modulus and strength values were based on the average of at least ten samples. A second type of specimen (125 mm × 125 mm plate) was used to characterize the impacted material based on the mean failure energy (MFE), according to the low-temperature impact test protocol established by the “Association of Rotational Molders International (ARM)” [74]. The MFE tests were carried out on a test rig constructed according to the drawings on pages 9–12 of the low-temperature impact test instructions of the “Association of Rotational Molders International (ARM)” [74] at minus 40 °C. The samples for the determination of MFE were kept at a temperature of minus (40 ± 1) °C for 24 h in a freezer. The reported values of MFE_sp._ are based on the average of at least 18 specimens and are given in J/mm, calculated using the following Formula (4):

(4) $$MFE_{sp.}=\frac{MFE}{S}$$

where S is the sample thickness, in mm;

MFE_sp._—specific mean failure energy, J/mm;

MFE—mean failure energy, J.

Sample density was measured based on the Archimedes method as described in the International Organization for Standardization (ISO) 1183 document using an electronic densimeter, model MD-300S, Japan, at room temperature and in distilled water.

### 2.5. Experimental Design

#### Planning an Experiment for Modeling

The simulation of the joint influence of the PIAT value, temperature, and thickness of the rotational plastic sample on the amplitude coefficient value of the third harmonic (β, r.u.a.) of the ultrasonic signal was carried out using the method of probabilistic–deterministic planning (PDP). A detailed description of the PDP method for modeling multiparameter processes is presented in [75,76,77,78,79,80,81]. The experiment was designed with a “3 × 3 × 4” plan and included all the possible combinations of input parameters. In this plan, PIAT and the thickness of the rotational plastic samples varied at three levels, and the temperature of the rotational plastic samples at four levels.

For modeling using the PDP method, the following actions were performed:

1. The following factors were identified as input parameters:
  - PIAT value, °C: for ELTEX, these values were 170, 200, and 235; for DOW, they were 170, 200, and 235; for M350, they were 180, 210, and 245;
  - The thickness of the plastic sample, S, mm: for all plastic samples, these values were set as 7.5, 8.5, and 9.5;
  - Sample temperature, T, °C: we used the following values: 20, 40, 60, and 80.
2. Furthermore, for each studied plastic sample, a “3 × 3 × 4” plan was designed. Table 2 presents the “3 × 3 × 4” plan designed for the experiment and the measurement values of the amplitude coefficient of the third harmonic in each experiment.

## 3. Results

### 3.1. Influence of the Temperature on Relative Speed of Ultrasonic Signal

**Figure 5:**
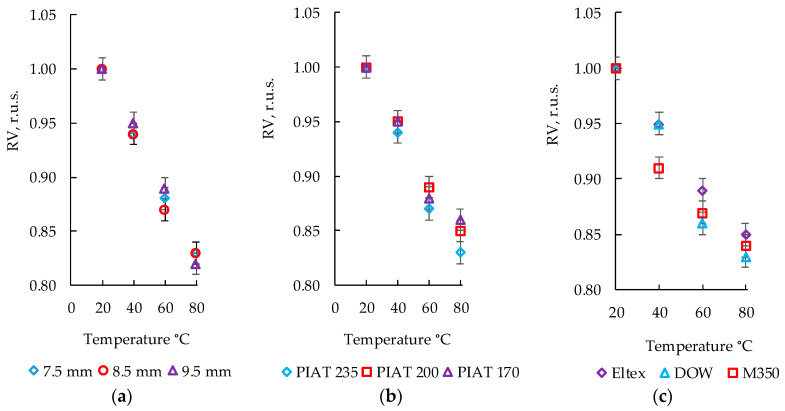
The effect of temperature on the relative ultrasonic signal transmission speed (RV, r.u.s.) with the echo-pulse method of control, for PE brand ELTEX PIAT 170 °C (**a**); for PE brand DOW thickness 8.5 mm (**b**); and for PE thickness 8.5 mm and normal-sintering specimens (**c**).

The influence of temperature on the relative speed of ultrasonic signal transmission (RV, r.u.s.) was analyzed in PE test samples (taking into account different thicknesses, PIAT values, and brands) using the echo-pulse method of control, and the results are shown in Figure 5.

### 3.2. Influence of PE Thickness and Brand on the Temperature Coefficient of Stability

**Figure 6:**
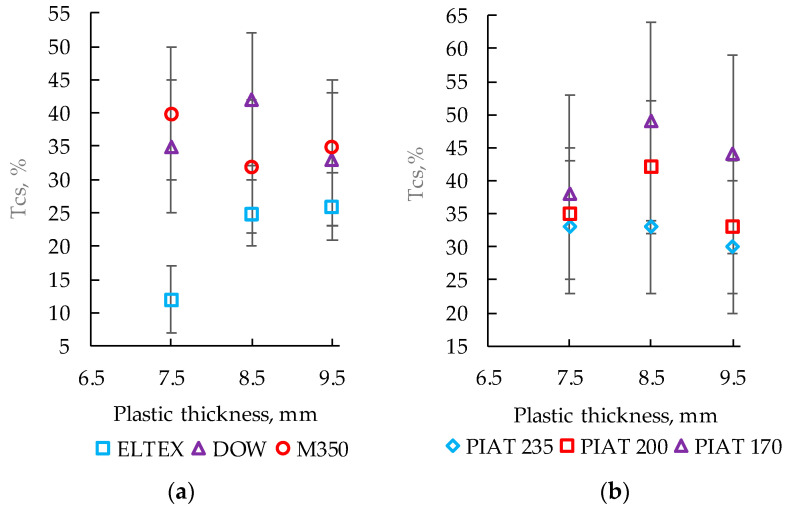
TCS for PE brands, ELTEX, DOW, and M350 (**a**);TCS for PE brand DOW with different PIAT values (**b**).

The effect of PE thickness and brand on the temperature coefficient of stability (TCS, %) is shown in Figure 6a. The influence of the sintering degree of sintering on TCS is shown in Figure 6b.

### 3.3. Effect of PE Quality on the Ultrasonic Signal Spectrum

**Figure 7:**
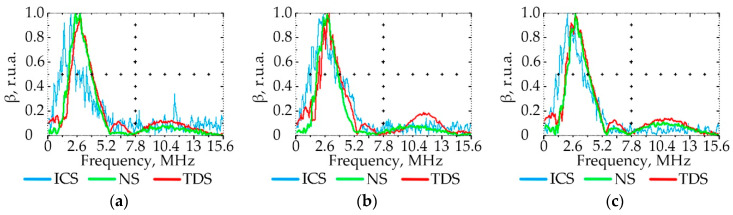
The amplitude coefficient of the third harmonic (β) at 20 °C for three sintering classes (ICS, NS, and TDS) for ELTEX (**a**), DOW (**b**), and M350 (**c**).

**Figure 8:**
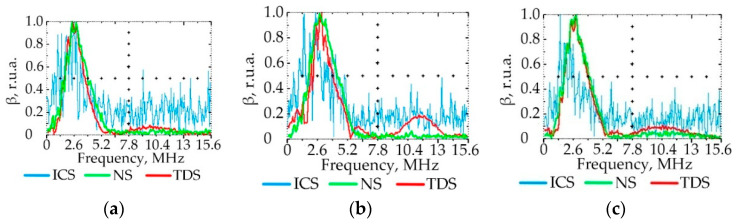
The amplitude coefficient of the third harmonic (β) at 40 °C for three sintering classes (ICS, NS, and TDS) for ELTEX (**a**), DOW (**b**), and M350 (**c**).

**Figure 9:**
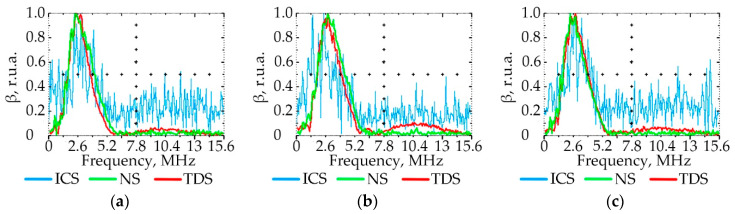
The amplitude coefficient of the third harmonic (β) at 60 °C for three sintering classes (ICS, NS, and TDS) for ELTEX (**a**), DOW (**b**), and M350 (**c**).

**Figure 10:**
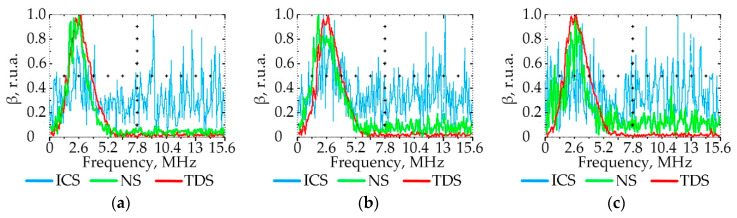
The amplitude coefficient of the third harmonic (β) at 80 °C for three sintering classes (ICS, NS, and TDS) for ELTEX (**a**), DOW (**b**), and M350 (**c**).

Figure 7, Figure 8, Figure 9 and Figure 10 show the graphs of the amplitude coefficient of the third harmonic (β) of the ultrasonic signal versus temperature for the three brands of plastic with a thickness of 8.5 mm. The effects of temperature on β for the ELTEX brand PE are shown in Figure 7a, Figure 8a, Figure 9a and Figure 10a, for DOW in Figure 7b, Figure 8b, Figure 9b and Figure 10b, and for M350 in Figure 7c, Figure 8c, Figure 9c and Figure 10c.

### 3.4. Influence of the Temperature on the Amplitude of the Bottom Ultrasonic Signal

**Figure 11:**
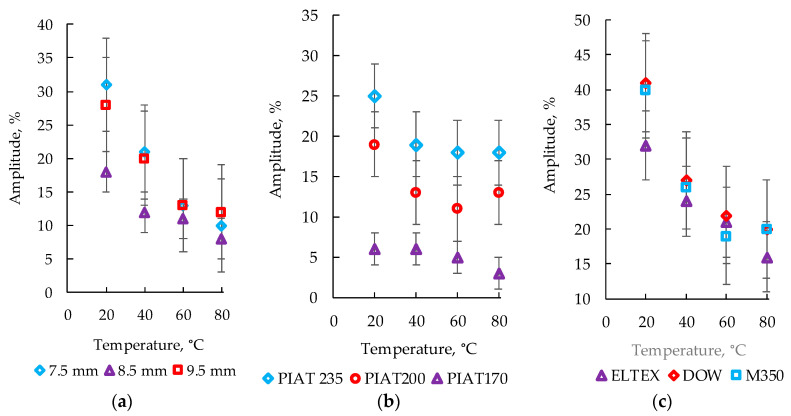
Graphs of the influence of the plastic heating temperature on the amplitude of the bottom signal during the echo-pulse method of control under conditions of different material thicknesses for PE brand M350 and PIAT 210 °C (**a**), different PIAT values for PE brand ELTEX with a thickness of 9.5 mm (**b**), and different PE brands with a thickness of 8.5 mm and “TDS” (**c**).

The effect of temperature, taking into account the thickness of the samples, as well as the brands of PE and PIAT, on the amplitude of the bottom signal (A) is shown in Figure 11.

### 3.5. Development of a Mathematical Model

**Figure 12:**
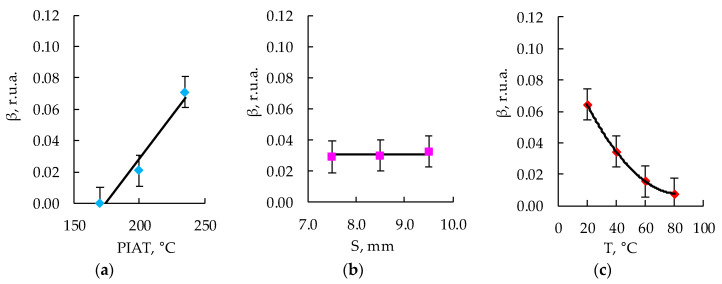
Influence of PIAT (**a**), thickness (S) (**b**), and temperature (T) (**c**) on the amplitude coefficient of the third harmonic (β) for ELTEX.

**Figure 13:**
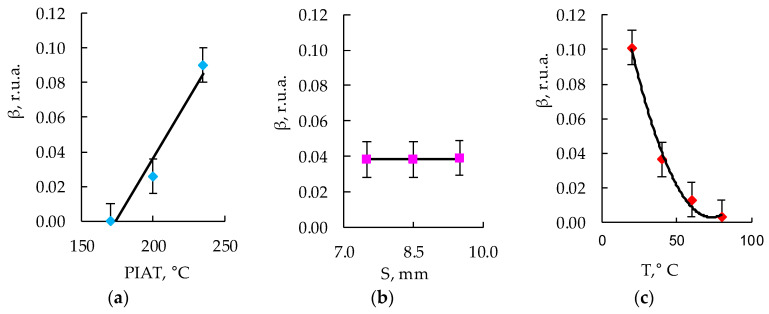
Influence of PIAT (**a**), thickness (S) (**b**), and temperature (T) (**c**) on the amplitude coefficient of the third harmonic (β) for DOW.

**Figure 14:**
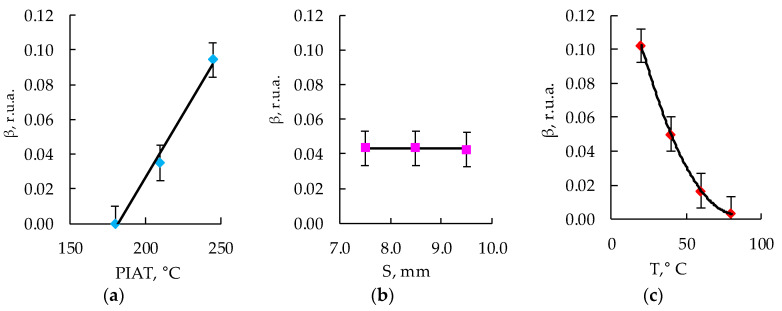
Influence of PIAT (**a**), thickness (S) (**b**), and temperature (T) (**c**) on the amplitude coefficient of the third harmonic (β) for M350.

After measuring the values of the amplitude coefficient of the third harmonic corresponding to a certain combination of input parameters (Table 2), the experimental array was sampled on dot plots in accordance with the PDP method [75,76,77,78,79,80,81], and the particular dependencies were obtained, which are shown in Figure 12, Figure 13 and Figure 14.

To derive a multifactorial statistical mathematical model of the effect of PIAT and temperature on β, the following equation proposed by M. M. Protodiakonov (5) was used:

(5) $$Y_{o}=\frac{\prod_{i=1}^{p}Y_{i}}{Y_{M}^{p-1}}$$

where Y_o_ is the generalized equation; Y_i_ is a particular function; $\prod_{i=1}^{p}Y_{i}$ is the product of all particular functions; p is the number of particular functions equal to the number of input parameters; and $Y_{M}^{p-1}$ is the general average of all the considered experimental values to a degree one less than the number of particular functions.

The reliability of the obtained mathematical model was determined by calculating the coefficient of nonlinear multiple correlations as follows:

(6) $$R=\sqrt{1-\frac{\left(n-1\right)\cdot \sum_{i=1}^{n}{\left(y_{i}-{yc}_{i}\right)}^{2}}{\left(n-p-1\right)\cdot \sum_{i=1}^{n}{\left(y_{i}-ym\right)}^{2}}}$$

where n is the number of experiments; p is the number of input (independent) parameters; i is the serial number of the experiment; y_i_ is the actual value of the output parameter in the i experiment; yc_i_ is the calculated value of the output parameter, calculated using a multifactor mathematical model, for the conditions (values of input parameters) of the i experiment; and ym is the average value of the actual value of the output parameter for all n experiments (the general average).

Using the obtained particular dependencies, shown in Figure 12, Figure 13 and Figure 14, a multifactor mathematical model (7) was built based on the generalized Protodyakonov Equation (5) as follows:

(7) $$\beta =\frac{\left(a+b\cdot PIAT\right)\cdot \left(c+d\cdot T+e\cdot T^{2}\right)}{\beta_{m}},r.u.a.$$

The thickness of the samples in the mathematical model was not taken into account, since during the experiments, it was found that there was no statistically significant effect of the thickness of the sample on the amplitude coefficient of the third harmonic (Figure 12b, Figure 13b and Figure 14b). Table 3 shows the values of the coefficients included in Equation (7) for the three different PE brands.

The reliability of the obtained mathematical models was estimated by calculating the coefficients of nonlinear multiple correlations (6). The minimum coefficient of nonlinear multiple correlations among the proposed mathematical models was 0.8.

### 3.6. Influence of PIAT on the Mechanical Properties of Samples

**Figure 15:**
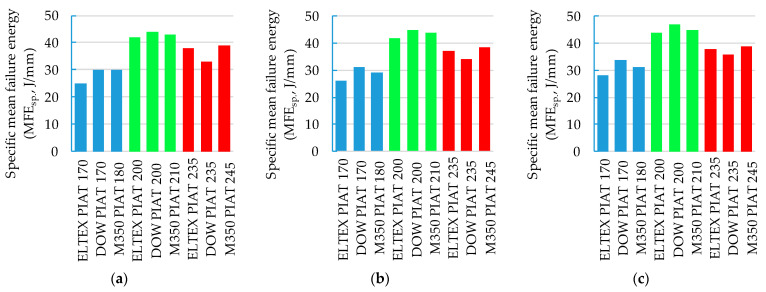
Influence of PIAT, PE brand, and sample thickness on specific mean failure energy (MFE_sp._, J/mm): (**a**) 7.5 mm; (**b**) 8.5 mm; (**c**) 9.5 mm.

The impact of PIAT on the specific mean failure energy (MFE_sp._, J/mm) is shown in Figure 15.

**Figure 16:**
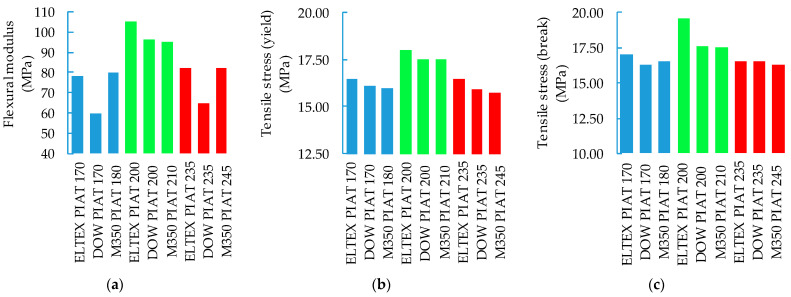
Influence of PIAT, PE brand, and sample thickness on the mechanical properties of PE samples: (**a**) 7.5 mm; (**b**) 8.5 mm; (**c**) 9.5 mm.

The effect of PIAT on the mechanical properties of PE samples is shown in Figure 16.

## 4. Discussion

Comparing the mechanical properties and the specific mean failure energy (MFE_sp._, J/mm) for the 27 samples, 9 of them with normal PIAT (No. 4, 5, 6, 13, 14, 15, 22, 23, and 24 from Table 1) were recognized as usable samples (US). Nine samples with minimal PIAT (No. 1, 2, 3, 10, 11, 12, 19, 20, and 21 from Table 1) and nine samples with maximum PIAT (No. 7, 8, 9, 16, 17, 18, 25, 26, and 27 from Table 1) were recognized to be wrong (WR).

The relative speed of ultrasound (RV, r.u.s.) decreased with an increase in the PE temperature (from 20 to 80 degrees Celsius) by 15–18%, regardless of the thickness (7.5 mm, 8.5 mm, or 9.5 mm) and the brand of plastic Figure 5a. For all three degrees of sintering quality (NS, TDS, and ICS), RV was in the range from 1 to 0.82, with an error of ±0.01 r.u.s. when the temperature changed from 20 to 80 °C. Similarly, RV decreased with the increasing temperature for PE with different degrees of sintering quality. It was not possible to identify statistically significant differences between samples with different degrees of sintering quality (i.e., to separate usable samples (US) from wrong (WR) ones) within the framework of the RV analysis. The effect of PE brands on RV was not revealed. It was not possible to identify a specific PE brand from the results of the ultrasonic inspection.

For ELTEX, depending on the PE thickness, TCS was between 13% and 26% (with an error of ±5%), as shown in Figure 6a. Samples DOW and M350 differed from ELTEX: for the first brand, TCS ranged from 33% to 42% (with an error of ±10%), while for the second brand, it ranged from 32% to 40% (with an error of ±10%). It was not possible to determine the quality category of PE through TCS analysis. No correlation was found between PIAT and TCS (Figure 6b).

As a result of the analysis of the amplitude coefficient of the third harmonic of the ultrasonic signal (β) (Figure 7, Figure 8, Figure 9 and Figure 10), the dependence of the attenuation of the third harmonic of the bottom signal on the quality of sintering was revealed, which was first identified in the work of Gomes and Thompson [68]. These authors showed that with an increase in PIAT, the attenuation of the third harmonic of the bottom signal decreases. This was confirmed in this study by the increase in β for the test samples with a large PIAT value during the transition between sintering classes “ICS”, “NS”, and “TDS”. It has been established that the amplitude coefficient β changes under conditions of different PE temperatures. This feature is typical for different brands of PE and different sintering classes. This dependence was confirmed by the results presented in Figure 7, Figure 8, Figure 9 and Figure 10. At a temperature of 20 °C, the amplitude coefficient β for the “TDS” degree was 0.2 ± 0.02 r.u.a., while for “NS”, this was 0.1 ± 0.02 r.u.a. For ICS samples, the appearance of the third harmonic was not typical. In this case, broadband noise was observed with a noise level (NN) of no more than 0.15 ± 0.02 r.u.a. As the temperature increased, β decreased for samples with “NS” and “TDS” degrees. For ICS samples, when heated from 20 °C to 80 °C, NN increased from 0.15 ± 0.02 r.u.a to 0.75 ± 0.02 r.u.a. With the increasing temperature, the ability to identify the quality of sintering through the parameters β and NN was retained. For “TDS” samples, the parameter β decreased at 40 °C to 0.1 r.u.a; in the case of 60 °C, β was equal to 0.05 ± 0.02 r.u.a. At a temperature of 80 °C, β was 0 r.u.a, and NN was used as the analytical parameter, which was 0.05 ± 0.02 r.u.a. For specimens with the “NS” degree, the β coefficient was always lower than for “TDS” specimens. At a heating temperature of 40 °C, β decreased to 0.02 r.u.a, and with an increase in temperature to 60 °C, the third harmonic in the frequency band 7.8 MHz–13.5 MHz was not observed, whereas noise was observed with a level of NN 0.05 ± 0.02 r.u.a. A further increase in temperature to 80 °C led to an increase in the NN noise to 0.1 ± 0.02 r.u.a. At high temperatures (60–80 °C), the ICS samples had the highest NN values, and the TDS samples had the lowest level. The thickness and brand of PE did not affect the identification of sintering quality through β and NN values.

Increasing the PE thickness did not affect the temperature dependence of the bottom signal amplitude A (Figure 11a). In this case, the maximum value of this parameter was observed with the heating temperature of 20 °C, which is shown in Figure 11a–c. As the temperature of PE increased, the value of A decreased nonlinearly. From a temperature of 60 °C, an increase in A was observed, but the peak value did not reach the level set at 20 °C. The A value for the partially sintered PE samples at the heating temperatures studied was much lower than that for the “TDS” and “NS” samples (Figure 11b). The A values for the “TDS” samples were higher than those for the “NS” samples (Figure 11b). However, taking into account the large error of values reaching 7%, this condition was not appropriate for the identification of the quality of sintering between “TDS” and “NS” samples. It was not possible to identify a specific brand of PE (out of the three studied) through the temperature dependence of A using the echo-pulse method of ultrasonic inspection control (Figure 11c).

As a result of the analysis of Figure 15 and Figure 16, it was possible to determine the degree of validity of the product (“US”—samples with the “NS” degree of sintering quality; “WR”— samples with the “ICS” and “TDS” degrees of sintering quality) using a traditional destructive control method based on MFE. The specific mean failure energy of “US” samples was over 40 J/mm (Figure 15a–c), the flexural modulus was over 90 MPa (Figure 16a), and their tensile stress was over 17.5 MPa (Figure 16b,c). This method of control is costly and inefficient in comparison with the ultrasonic method.

**Figure 17:**
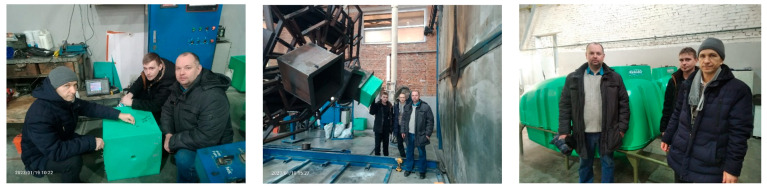
Testing of the proposed ultrasonic inspection method on the AVAGRO LLP facility.

To assess the prospects of introduction in the industry and embedding the developed method of ultrasonic inspection for the quality control of plastic products into the existing technological process of rotational molding, tests were carried out at the AVAGRO LLP facility. This organization is the largest manufacturer of tanks for agrotechnological purposes (the storage and transportation of pesticide solutions and liquid mineral fertilizers) in the Republic of Kazakhstan. Photographs of ultrasonic inspection tests are shown in Figure 17.

**Figure 18:**
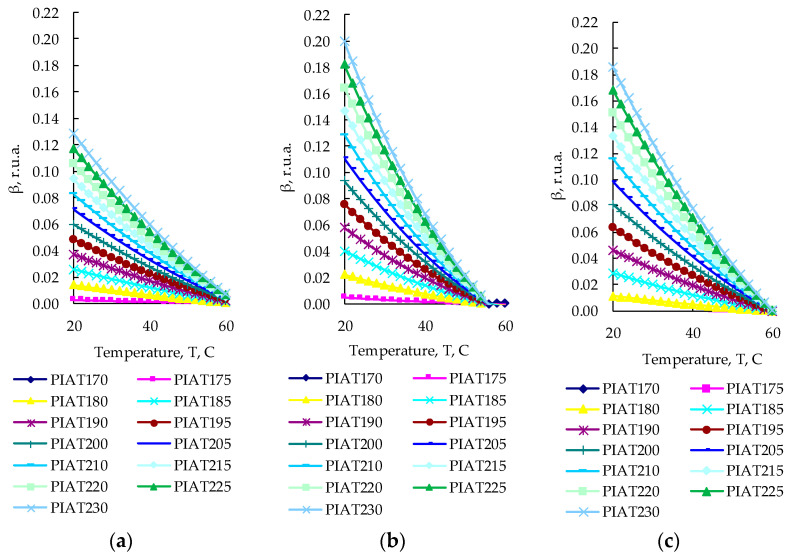
Two-factor nomograms: (**a**) β = *f* (T, PIAT) for ELTEX; (**b**) β = *f* (T, PIAT) for DOW; (**c**) β = *f* (T, PIAT) for M350.

Based on the analysis results of ultrasonic parameters (A, RV, TCS, and β), only the amplitude of the third harmonic (β) demonstrated the ability to determine the sintering classes “ICS”, “NS”, and “TDS”. It was decided to use the mirror-shadow method of measuring β to identify sintering classes and to identify the “WR” and “US” PE products. The preliminary tests of the proposed ultrasonic inspection method were quite encouraging, so the accuracy of determining the “ICS”, “NS”, and “TDS” degrees by analyzing the amplitude coefficient of the third harmonic of the ultrasonic signal (β) on the manufactured products was at least 95%. At the request of the technological service of AVAGRO LLP, in order to eliminate complex and cumbersome calculations, three nomograms were developed, which are presented in Figure 18.

These nomograms enable the quick determination of the degree of sintering quality (“ICS”, “NS”, and “TDS”) of a particular product from the obtained experimental value, namely the amplitude coefficient of the third harmonic of the ultrasonic signal (β), taking into account the actual temperature of the tank. In this case, it is always possible to compare the experimentally obtained PIAT with that obtained from the nomogram, which potentially helps to identify failures in the operation of control and measuring equipment (thermocouples, etc.).

Due to the complexity of solving the problem of embedding the proposed ultrasonic inspection method into the rotational molding process, a logical continuation of this study is the development of automatic algorithms (possibly with the involvement of neural networks) that would identify the degree of sintering quality (“ICS”, “NS”, and “TDS”) of a particular product in automatic mode, according to the results of measurements of ultrasonic parameters.

The introduction of the developed method of ultrasonic inspection into the industry will facilitate the quality control of all tanks manufactured at the enterprise. This will not only significantly increase the reputation of the company but will also prevent potential toxic pesticides from entering the soil and poisoning groundwater in case of the destruction of tanks.

## 5. Conclusions

(1) It was shown that the thickness of the walls of PE tanks did not statistically significantly affect the studied parameters of the ultrasonic signal: the temperature coefficient of stability (TCS), the relative velocity (RV), the amplitude coefficient of the third harmonic (β), and the noise level (NN). As the thickness increased, the amplitude of the bottom signal (A) decreased, but the value was recorded with a large error. This significant error in measuring the amplitude of the bottom signal was due to the fact that the samples obtained under the conditions of industrial rotomolding did not have plane-parallel walls. Moreover, the deviation from parallelism varied even within the borders of every sample, which made it difficult to obtain reproducible results when measuring the amplitude of the bottom signal. Therefore, the thickness factor can be ignored during the ultrasonic inspection of PE containers manufactured via rotomolding.
(2) The influence of the PE temperature on all the investigated parameters of the ultrasonic signal (A, TCS, RV, β, and NN) was proven. As the temperature increased, RV equally decreased for PE with different degrees of sintering quality (NS, TDS, and ICS). By analyzing the temperature coefficient of stability (TCS), it was possible to distinguish the ELTEX brand of plastics from DOW and M350. With increasing temperature, A decreased nonlinearly.
(3) The possibility of determining the degree of quality of PE sintering through the analysis of the amplitude of the third harmonic of the ultrasonic signal (β) using the mirror-shadow method was confirmed. At low temperatures ranging from 20 to 40 °C, the PE sintering quality grades (NS, TDS, and ICS) were identified by β: for “NS”, β = 0.1 ± 0.02; for “TDS”, β = 0.2 ± 0.02; and for “ICS”, β = 0. At medium temperatures ranging from 40 to 60 °C, for “NS”, β = 0.05 ± 0.02; for “TDS”, β = 0.1 ± 0.02; and for “ICS”, β = 0. At high temperatures (60–80 °C), the third harmonic was not found. At such temperatures, it was necessary to use the amplitude noise factor (NN): for “ICS”, NN = 0.75 ± 0.02; for “NS”, NN = 0.1 ± 0.02; and for “TDS”, NN = 0.05 ± 0.02. The thickness and brand of PE did not affect the possibility of identifying the quality of sintering through β and NN.

As a result of the tests, β was chosen as the most informative characteristic capable of revealing the degree of sintering quality, with an accuracy of at least 95%. The equations β = *f*(T, PIAT) were derived for each brand of the studied PE, and two-factor nomograms were constructed. These nomograms facilitate the rapid identification of the sintering class “ICS”, “NS”, and “TDS” of a particular product according to the experimental value of β, taking into account the actual temperature of the tank and without complex calculations.

(4) In the studies of other authors on this topic, as a rule, frequencies ranging from 150 to 400 kHz were used. For this frequency range, more expensive specialized “low-frequency” ultrasonic flaw detectors are required. In this research, a standard ultrasonic flaw detector was used, which is usually used for metal product inspection. The studies were carried out at frequencies ranging from 2.5 to 3.75 MHz. Since it is difficult to carry out an ultrasonic inspection at a strictly fixed temperature under industrial conditions, the authors calculated and constructed special nomograms. These nomograms enable inspection at any temperature ranging from 20 to 40 °C, followed by the determination of the sintering quality of the PE product, taking into account the actual temperature. Thus, an ultrasonic inspection method was developed, and semi-industrial tests were carried out at an industrial enterprise, which confirmed its promise. The results showed a detection accuracy of the sintering quality levels of at least 95%.

## Figures and Tables

**Table 1:** Quality categories of PE samples.

Sample Number	Product Density, g/cm^3^	Sample Thickness, mm	PIAT, °C	Sample Material Brand	Specific Mean Failure Energy (MFE_sp._), J/mm	The Degree of Quality of Sintering Products, (NS, ICS, and TDS)	The Degree of Validity of the Product, US/WR
1	0.932	7.5	170	ELTEX	25	ICS	WR
2	0.932	7.5	170	DOW	30	ICS	WR
3	0.933	7.5	180	M350	30	ICS	WR
4	0.940	7.5	200	ELTEX	42	NS	US
5	0.942	7.5	200	DOW	44	NS	US
6	0.936	7.5	210	M350	43	NS	US
7	0.943	7.5	235	ELTEX	38	TDS	WR
8	0.944	7.5	235	DOW	33	TDS	WR
9	0.945	7.5	245	M350	39	TDS	WR
10	0.932	8.5	170	ELTEX	26	ICS	WR
11	0.932	8.5	170	DOW	31	ICS	WR
12	0.933	8.5	180	M350	29	ICS	WR
13	0.942	8.5	200	ELTEX	42	NS	US
14	0.943	8.5	200	DOW	45	NS	US
15	0.938	8.5	210	M350	44	NS	US
16	0.944	8.5	235	ELTEX	37	TDS	WR
17	0.945	8.5	235	DOW	34	TDS	WR
18	0.945	8.5	245	M350	38.5	TDS	WR
19	0.932	9.5	170	ELTEX	28	ICS	WR
20	0.933	9.5	170	DOW	34	ICS	WR
21	0.933	9.5	180	M350	31	ICS	WR
22	0.940	9.5	200	ELTEX	44	NS	US
23	0.943	9.5	200	DOW	47	NS	US
24	0.944	9.5	210	M350	45	NS	US
25	0.945	9.5	235	ELTEX	38	TDS	WR
26	0.945	9.5	235	DOW	36	TDS	WR
27	0.945	9.5	245	M350	39	TDS	WR

**Table 2:** The “3 × 3 × 4” experiment plan designed to study the effect of PIAT, temperature, and sample thickness on the amplitude coefficient of the third harmonic of the ultrasonic signal for three different rotational plastic specimens.

n	PIAT, °C			Thickness, S, mm	Temperature, T, °C	Amplitude Coefficient of the Third Harmonic, β, r.u.a.		
	ELTEX	DOW	M350			ELTEX	DOW	M350
1	235	235	245	7.5	20	0.12	0.21	0.21
2	235	235	245	7.5	40	0.10	0.10	0.11
3	235	235	245	7.5	60	0.04	0.04	0.05
4	235	235	245	7.5	80	0.01	0.01	0.01
5	235	235	245	8.5	20	0.12	0.21	0.21
6	235	235	245	8.5	40	0.09	0.10	0.11
7	235	235	245	8.5	60	0.05	0.04	0.05
8	235	235	245	8.5	80	0.01	0.01	0.01
9	235	235	245	9.5	20	0.12	0.21	0.20
10	235	235	245	9.5	40	0.09	0.10	0.11
11	235	235	245	9.5	60	0.05	0.04	0.05
12	235	235	245	9.5	80	0.05	0.01	0.01
13	200	200	210	7.5	20	0.07	0.09	0.10
14	200	200	210	7.5	40	0.01	0.01	0.04
15	200	200	210	7.5	60	0.00	0.00	0.00
16	200	200	210	7.5	80	0.00	0.00	0.00
17	200	200	210	8.5	20	0.08	0.09	0.10
18	200	200	210	8.5	40	0.01	0.01	0.04
19	200	200	210	8.5	60	0.00	0.00	0.00
20	200	200	210	8.5	80	0.00	0.00	0.00
21	200	200	210	9.5	20	0.07	0.10	0.10
22	200	200	210	9.5	40	0.01	0.01	0.04
23	200	200	210	9.5	60	0.00	0.00	0.00
24	200	200	210	9.5	80	0.00	0.00	0.00
25	170	170	180	7.5	20	0.00	0.00	0.00
26	170	170	180	7.5	40	0.00	0.00	0.00
27	170	170	180	7.5	60	0.00	0.00	0.00
28	170	170	180	7.5	80	0.00	0.00	0.00
29	170	170	180	8.5	20	0.00	0.00	0.00
30	170	170	180	8.5	40	0.00	0.00	0.00
31	170	170	180	8.5	60	0.00	0.00	0.00
32	170	170	180	8.5	80	0.00	0.00	0.00
33	170	170	180	9.5	20	0.00	0.00	0.00
34	170	170	180	9.5	40	0.00	0.00	0.00
35	170	170	180	9.5	60	0.00	0.00	0.00
36	170	170	180	9.5	80	0.00	0.00	0.00

**Table 3:** The values of the coefficients included in Equation (7) for the three different PE brands.

PE Brand	a	b	c	d	e	β_m_
ELTEX	−0.1911	0.0011	0.1056	−0.0023	0.00001	0.0306
DOW	−0.2431	0.0014	0.1858	−0.005	0.00003	0.0386
M350	−0.2650	0.0015	0.1742	−0.0041	0.00002	0.0431

## Data Availability

The datasets generated during and/or analyzed during the current study are available from the corresponding author upon reasonable request.
